# Robot-Assisted Radical Cystectomy for Invasive Bladder Cancer With Vesicovaginal Fistula: A Case Report

**DOI:** 10.7759/cureus.65234

**Published:** 2024-07-24

**Authors:** Risako Watanabe, Jun Kamei, Masahiro Yamazaki, Toru Sugihara, Tetsuya Fujimura

**Affiliations:** 1 Urology, Jichi Medical University Hospital, Tochigi, JPN; 2 Urology, The University of Tokyo Hospital, Tokyo, JPN

**Keywords:** sacrocolpopexy, radical cystectomy, vesicovaginal fistula, robotic surgery procedures, bladder cancer

## Abstract

An 89-year-old woman was diagnosed with vesicovaginal fistula (VVF) during transurethral surgery for repeated bladder cancer recurrences. She was referred to our hospital for the treatment of VVF and pT1 invasive bladder cancer. Typical radical cystectomy procedures incising the vaginal wall are not suitable for her because of the high risk of disseminating tumors. Robot-assisted radical cystectomy with *en bloc* resection of the bladder, uterus, ovaries, and vagina was successfully performed without urine extravasation by dissecting the rectovaginal space to the pelvic floor, referencing the robot-assisted sacrocolpopexy technique. No evidence of recurrence was noted within 10 months after surgery.

## Introduction

Bladder cancer is one of the most common urogenital tract cancers, which are usually classified into non-muscle invasive (≤T1) and muscle invasive (≥T2) bladder cancer. Standard treatment for non-muscle invasive bladder cancer is transurethral surgery with or without intravesical therapy and that for muscle invasive or high-risk T1 bladder cancer is radical cystectomy [1,2]. Robot-assisted radical cystectomy (RARC) has become a standard minimally invasive surgery, which reduces perioperative complications with comparable oncological outcomes to open surgery [3]. The uterus, ovaries, and anterior vaginal wall are usually resected with the bladder in RARC in female patients [4]. 

Vesicovaginal fistula (VVF) is an abnormal communication between the bladder and vagina, resulting in continuous urine leakage into the vagina. VVF can be caused by several factors such as childbirth, cesarean section, hysterectomy, trauma, and radiotherapy but VVF caused by bladder tumor is rare [5-8]. In patients with invasive bladder cancer with VVF, the risk of disseminating tumors to incise the vaginal wall or to dissect the vesicovaginal space is high, and it would be unsuitable for these patients to undergo RARC using typical surgical methods. Herein, we report a case of invasive bladder cancer with VVF treated with RARC with en bloc resection of the bladder, uterus, and both anterior and posterior vaginal walls by dissecting the rectovaginal space and pelvic floor.

## Case presentation

An 89-year-old woman diagnosed with a pT1 bladder tumor that cannot be resected adequately because of VVF presented to our department for treatment of both VVF and invasive bladder cancer. She received a right radical nephroureterectomy for right ureteral cancer at 83 years old and transurethral resection of a bladder tumor (TURBT) for a bladder tumor at 85 years old at another hospital. The histopathological diagnosis was high-grade pT1 urothelial carcinoma with carcinoma in situ (CIS), and intravesical Bacillus Calmette-Guérin (BCG) therapy was administered. Bladder cancer recurred frequently, and TURBT was performed each time the recurrence was confirmed. During the fifth TURBT, the VVF was identified and sutured transvaginally, and the fistula recurred shortly. Five months later, bladder cancer recurred; however, the sixth TURBT was not performed successfully because the bladder could not be expanded because of saline leakage from the VVF. The histopathological diagnosis was high-grade pT1 urothelial carcinoma, and, therefore, she was referred to our institute for treatment of both VVF and invasive bladder cancer.

**Figure 1:**
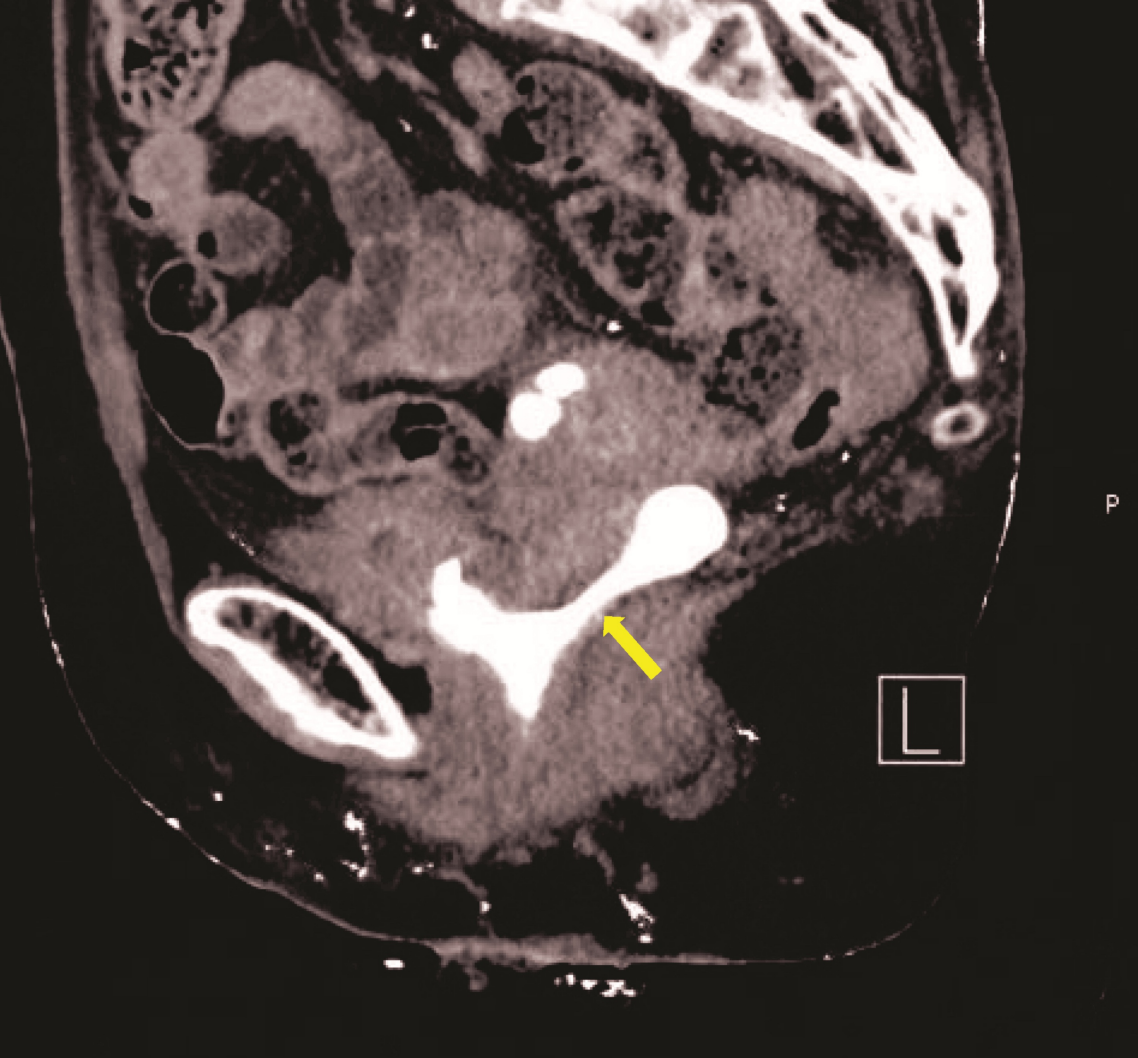
Excretory phase images of the contrast-enhanced CT. The contrast agent was stored from the bladder trigone into the vagina (arrow), suggesting a VVF. CT, computed tomography; VVF, vesicovaginal fistula

Contrast-enhanced computed tomography (CT) revealed no evidence of distant metastasis, and excretory phase images showed storage of the contrast agent in the vagina via the VVF at the bladder trigone (Figure 1).

After discussions within our department, we planned to perform RARC with en-bloc resection of the bladder, uterus, ovaries, and vagina, including the VVF, to avoid the intraperitoneal extravasation of urine during surgery. Neoadjuvant chemotherapy was not performed considering the short time from the first visit to the surgery and the pT1 bladder tumor with advanced age. 

**Figure 2:**
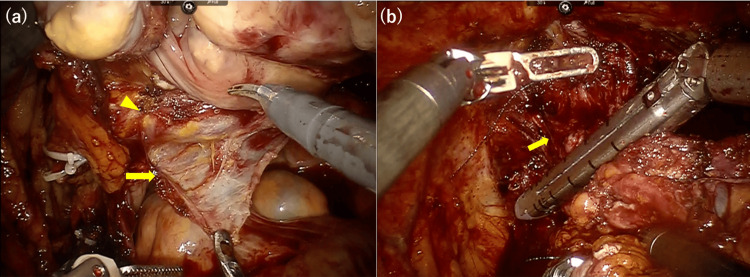
Intraoperative finding. (a) Dissecting between the posterior vaginal wall (arrowhead) and rectum from the Douglas fossa (arrow) with insertion of a 50 mm width intestinal spatula into the vagina. (b) Isolating the circumferentially dissected vagina (arrow) using endo-GIA at the distal side of the VVF. VVF, vesicovaginal fistula

The surgical procedure was performed with robotic assistance using the da Vinci Si system. After setting the patients in lithotomy position and placing six ports, the robot was docked in a 25° Trendelenburg position [9,10]. The left ureter was divided close to the ureterovesical junction, and the negative distal ureteric margin was confirmed. With the insertion of a 50 mm width intestinal spatula into the vagina, the rectovaginal space was dissected from the Douglas fossa to the fascia of the levator ani, referencing the robot-assisted sacrocolpopexy technique for pelvic organ prolapse [11]. The lateral pedicles were divided, and both lateral spaces of the vagina were dissected to the fascia of the levator ani. For lateral dissection of the vagina, the 50 mm width intestinal spatula was too large to recognize the shape of the vagina and the correct position of ligaments to be excised, and to ensure adequate space, we changed the intravaginal intestinal spatula to a thicker one (35 mm width) that can provide adequate traction and space keeping the shape of vagina relatively well. A peritoneal incision was made, and the Retzius space was entered. The dissected urethra was incised after ligation, and the circumferentially dissected vaginal wall remained intact. The vagina was isolated using a laparoscopic linear stapler (60 mm, endo-GIA) (Figure 2), and the bladder, uterus, ovaries, and vagina were detached without urine extravasation into the peritoneal cavity (Video 1).

**Video 1:** Video played at the triple speed of robot-assisted radical cystectomy for a female patient with VVF, avoiding opening the vagina or fistula to the peritoneal cavity. VVF, vesicovaginal fistula

Cutaneous ureterostomy was decided on for urinary diversion, and lymph node dissection was not performed to minimize surgical invasion, considering the age of the patient with cT1 bladder cancer. The operating and console time were 277 and 189 min, respectively, with 200 mL of blood loss.

**Figure 3:**
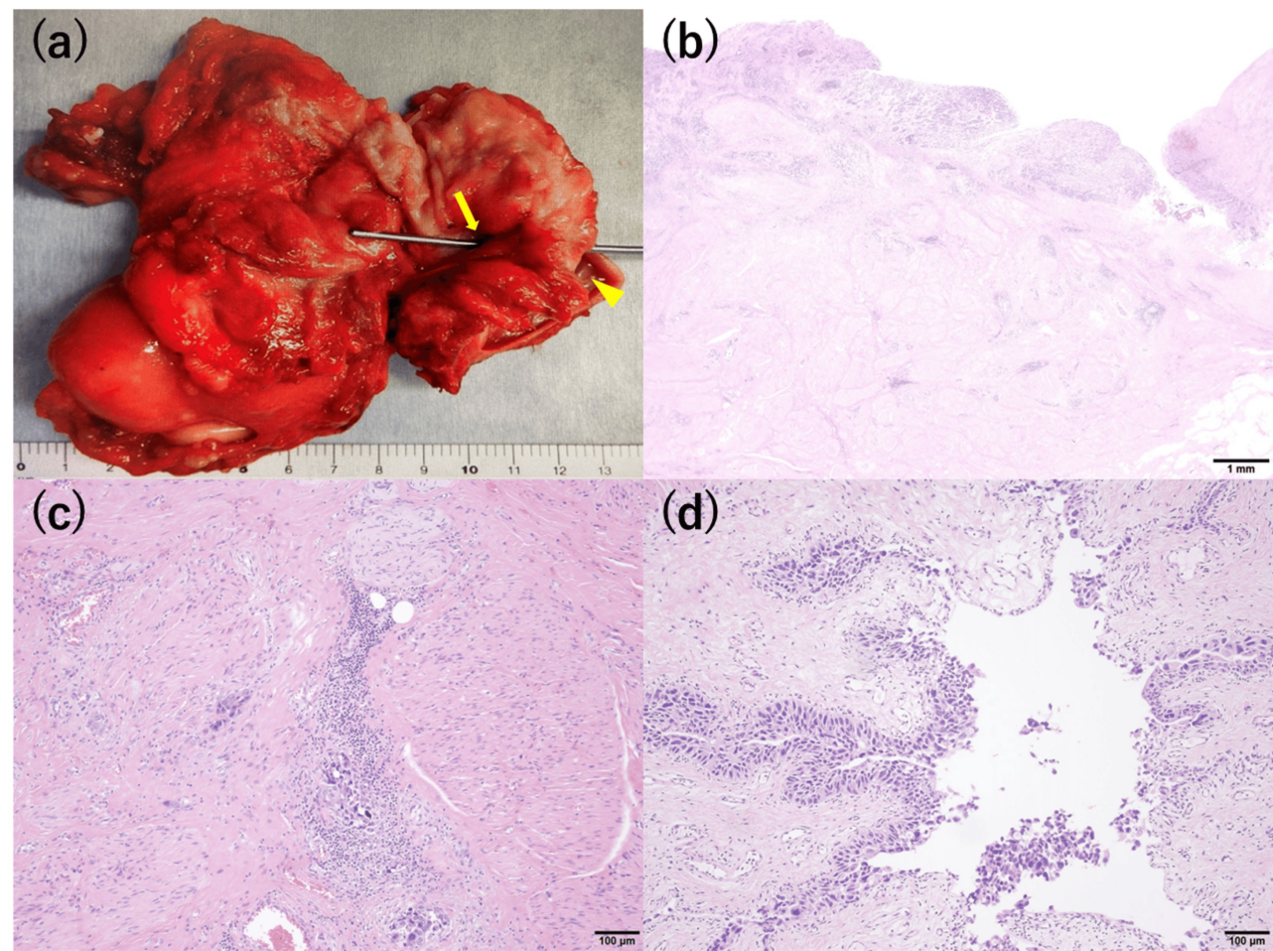
Macroscopic findings of the specimen after opening the bladder dome (a) and histopathological findings of the bladder (b, c) and left ureter (d). (a) A sonde was inserted into the fistula (arrow) from the bladder trigone to the vagina (arrowhead). (b, c) Hematoxylin and eosin staining of the bladder shows tumor cells of high-grade urothelial carcinoma scattered within the muscle layer and some extend to the serosal layer. (d) CIS is observed in the resected left ureter. CIS, carcinoma in situ

Macroscopically, a fistula in the bladder trigone to the vagina was observed, and the histopathological diagnosis was high-grade pT2b urothelial carcinoma of the bladder and CIS of the resected left ureter with negative surgical margin; however, no cancerous invasion to the uterus and vagina was observed (Figure 3).

The patient was discharged on postoperative day 11 without any major complications. Regular follow-up with CT and urine cytology every two to three months were performed and no recurrence or postoperative complications were observed for 10 months.

## Discussion

The major causes of VVF include cesarean section, other pelvic surgeries, delivery failure, and radiation therapy [8]. Moreover, transurethral surgery for bladder cancer was reported as the rare etiology of VVF [6,7]. Although it is not a common etiology, VVF after repeated TURBT could increase in the future as the aging population increases. Nevertheless, there have been no previous reports or established treatment strategies for bladder cancer with VVF. To the best of our knowledge, this is the first case of successful RARC for invasive bladder cancer with VVF by en bloc resection of the bladder, uterus, ovaries, and vagina and dissection of the rectovaginal space.

The standard treatment for pT1 bladder cancer is intravesical BCG treatment or RARC; however, BCG treatment is not suitable because of the inability of the bladder to store BCG [12]. RARC in standard surgical methods, such as incising the vaginal wall or dissecting the vesicovaginal space, was unsuitable for her owing to the risk of disseminating tumors with intraperitoneal extravasation of tumor cells with urine. In contrast, performing transvaginal or transabdominal VVF repair before treatment for bladder tumors has risks of tumor progression and disseminating tumors by the extravasation of tumor cells with urine during VVF repair.

Therefore, en bloc resection of the bladder, uterus, ovaries, and vagina is required to treat VVF and invasive bladder cancer without extravasation of urine. We believe that our treatment strategy was appropriate because no major perioperative or postoperative complications nor evidence of recurrence were observed within 10 months.

RARC can be performed without urine extravasation by circumferentially dissecting the vaginal wall around the urethra on the distal side of the VVF. Referencing the surgical technique of dissecting the rectovaginal space in robot-assisted sacrocolpopexy and obtaining the surgical technique for robot-assisted sacrocolpopexy enabled us to successfully perform RARC [13].

## Conclusions

Typical radical cystectomy procedures involving the vaginal wall are not suitable for invasive bladder cancer with VVF in women. RARC with en bloc resection of the bladder, uterus, ovaries, and vagina to avoid intraoperative extravasation of urine was successfully performed in a female patient with invasive bladder cancer and VVF. This surgical procedure, referencing the surgical technique of dissecting the rectovaginal space in robot-assisted sacrocolpopexy, may be an appropriate treatment strategy for females with invasive bladder cancer with VVF.
